# Delivery of Home and Community Based Services During a Pandemic: Unexpected Consequences

**DOI:** 10.1093/geroni/igab046.1793

**Published:** 2021-12-17

**Authors:** Emily Cherlin, Adeola Ayedun, Jane Straker, Traci Wilson, Amanda Brewster, Chris Rubeo, Leslie Curry

**Affiliations:** 1 Yale School of Public Health, Yale Global Health Leadership Initiative, New Haven, Connecticut, United States; 2 Yale School of Public Health, New Haven, Connecticut, United States; 3 Miami University, Oxford, Ohio, United States; 4 USAging, Washington, District of Columbia, United States; 5 University of California, Berkeley School of Public Health, Berkeley, California, United States

## Abstract

The COVID-19 pandemic required AAAs to pause essential services, serving as a catalyst for innovation. We examined such innovations as part of an explanatory mixed-methods, positive deviance study of AAA partnerships with health and social service organizations. We identified 8 AAAs with many partners serving areas with lower levels of health care use, and 3 AAAs with few partners serving areas with higher levels of health care use. We interviewed AAA and partners, (total = 123). Using the constant comparative method, we identified recurrent themes: 1) AAAs adapted to increased demand for services by developing new ways to deliver services, 2) the pandemic raised awareness of unmet needs such as social connection, 3) changes in delivery of services included embracing technology, and 4) AAAs and their partners identified resources to rapidly pivot services. AAAs and partners demonstrated resiliency to not only to sustain programs, but to innovate throughout the pandemic.

